# Insomnia increases the risk for specific autoimmune diseases: a large-scale retrospective cohort study

**DOI:** 10.3389/fnetp.2025.1499297

**Published:** 2025-04-10

**Authors:** Sarah Stenger, Artem Vorobyev, Katja Bieber, Tanja Lange, Ralf J. Ludwig, Jennifer E. Hundt

**Affiliations:** ^1^ Lübeck Institute of Experimental Dermatology, University of Lübeck, Lübeck, Germany; ^2^ Department of Dermatology, University Hospital Schleswig-Holstein Lübeck, Lübeck, Germany; ^3^ Department of Rheumatology and Clinical Immunology, University Hospital Schleswig-Holstein Lübeck, Lübeck, Germany

**Keywords:** sleep, insomnia, autoimmunity, autoimmune diseases, TriNetX, retrospective cohort study

## Abstract

**Objective:**

The global rise of autoimmune diseases presents a significant medical challenge, with inadequate treatment options, high morbidity risks, and escalating healthcare costs. While the underlying mechanisms of autoimmune disease development are not fully understood, both genetic predispositions and lifestyle factors, particularly sleep, play critical roles. Insomnia and circadian rhythm sleep disorders not only impair sleep but also disrupt multi-organ interactions by dysregulating sympathetic nervous system activity, altering immune responses, and influencing neuroendocrine function. These disruptions can contribute to immune system dysregulation, increasing the risk of autoimmune disease development.

**Methods:**

To assess the impact of impaired sleep on the risk of developing autoimmune diseases, a global population-based retrospective cohort study was conducted using electronic health records from the TriNetX US Global Collaborative Network, including 351,366 subjects in each propensity score matched group. Twenty autoimmune diseases were examined, and propensity score matching was employed to reduce bias. Three sensitivity analyses were conducted to test the robustness of the results.

**Results:**

The study identified significantly increased risks for several autoimmune diseases associated with impaired sleep, likely mediated by dysregulated neuroimmune and autonomic interactions. Specifically, cutaneous lupus erythematosus [hazard ratio (HR) = 2.119; confidence interval (CI) 1.674–2.682; p < 0.0001], rheumatoid arthritis (HR = 1.404; CI 1.313–1.501; p < 0.0001), Sjögren syndrome (HR = 1.84; CI 1.64–2.066; p < 0.0001), and autoimmune thyroiditis (HR = 1.348; CI 1.246–1.458; p < 0.0001) showed significantly increased risks. No diseases demonstrated reduced risks, and 4 out of 20 tested diseases did not show significant HR increases in any analysis.

**Conclusion:**

This study highlights the integral role of sleep in maintaining immune homeostasis through multi-organ interactions involving the autonomic nervous system, immune signalling pathways, and endocrine regulation. Disruptions in these systems due to chronic sleep impairment may predispose individuals to autoimmune diseases by altering inflammatory responses and immune tolerance. These findings underscore the necessity of recognizing and treating sleep disorders not only for general wellbeing but also as a potential strategy to mitigate the long-term risk of autoimmune disease development.

## Introduction

Insomnia and circadian rhythm sleep disorders (CRSD) are common complaints among patients. Approximately 30% of the general population experiences chronic insomnia (Roth, 2007; Bhaskar et al., 2016). Women and older adults are more prone to insomnia, though the reasons for this disparity remain unclear. Hormonal changes associated with menstruation and menopause may contribute to increased prevalence in females (Johnson et al., 2006). Other potential causes of insomnia include stress, jet lag, shift work, environmental factors such as noise and light exposure, temperature fluctuations, psychiatric disorders, and genetic factors. While some of these factors may induce temporary sleep disturbances, approximately 40% of diagnosed insomnia cases become chronic and persistent (Sateia, 2014). CRSD affects around 3% of the population, but confusion with insomnia likely leads to underestimation, with estimates closer to 10% in adults (Kim et al., 2013). CRSD involves disruptions to the natural sleep-wake cycle, stemming from intrinsic factors like genetically advanced or delayed circadian clocks, or external influences such as shift work or jet lag. Symptoms of these disorders include daytime sleepiness, fatigue, mood swings, pain, physical discomfort, social functioning problems, and issues with concentration and memory, all of which can significantly impact quality of life (Roth, 2007). Moreover, these symptoms increase the risk of accidents and decrease work productivity (Léger et al., 2002; Kuppermann et al., 1995). Treating insomnia and CRSD can be challenging and may require lifestyle changes and psychotherapy. It is crucial to address these sleep disorders promptly, as prolonged insufficient sleep can lead to hypertension, insulin resistance (Shah et al., 2022), mood disorders, depression (Singareddy et al., 2012), anxiety, dementia (Jee et al., 2020), substance abuse (Roth and Workshop Participants, 2009), and impaired reaction times, increasing the risk of accidents (Inoue and Komada, 2014; Kalsi et al., 2018).

Beyond these well-established consequences, insomnia and CRSD may also disrupt multi-organ interactions, influencing the autonomic nervous system, immune regulation, and neuroendocrine signalling (Kim et al., 2022; Garbarino et al., 2021). Chronic sleep impairment has been shown to increase sympathetic nervous system activity, disrupt hypothalamic-pituitary-adrenal (HPA) axis function (Balbo et al., 2010), and alter immune responses (Garbarino et al., 2021), all of which may contribute to the onset and progression of autoimmune diseases. As demonstrated in this study, insomnia may trigger adverse immune reactions, potentially leading to autoimmunity and/or autoimmune diseases. The current understanding of the relationship between impaired sleep and autoimmunity suggests that it is primarily autoimmunity that leads to sleep disturbances (Iranzo, 2020). However, the potential risk of impaired sleep contributing to the development of autoimmune diseases has not been thoroughly investigated.

The risk of sleep disturbances in patients with autoimmune diseases is particularly well-documented for neurological autoimmune disorders such as multiple sclerosis and myasthenia gravis, where autoimmune damage to the brainstem and hypothalamus directly disrupts sleep regulation (Pérez-Carbonell and Iranzo, 2023). Another study investigated sleep in rheumatic diseases but primarily focused on the effects of these diseases on sleep, such as the influence of pain and decreased physical activity (Boeselt et al., 2019).

Hsiao and colleagues examined the effects of non-apnoea sleep disorders on autoimmune disease development using the National Health Insurance Research Database from Taiwan, demonstrating an increased risk of developing conditions such as systemic lupus erythematosus (SLE), rheumatoid arthritis (RA), Sjögren syndrome, and ankylosing spondylitis (Hsiao et al., 2015). Given that individuals with sleep disorders often have a history of mental disorders (MDs), it is essential to consider whether mental health conditions, rather than sleep disorders themselves, contribute to autoimmunity due to their known effects on the immune and stress systems. Previous studies had relatively small sample sizes, employed limited matching methods, and did not account for the influence of mental disorders, despite their known impact on autoimmunity (Kridin et al., 2022).

To clarify the role of insomnia in the development of autoimmune diseases, we utilized the TriNetX database, which provides access to a large repository of electronic health records. This allowed for the examination of even rare autoimmune diseases in sufficiently large sample sizes. Moreover, the use of advanced cohort matching techniques combined with sensitivity analyses enabled us to account for the influence of MDs and corresponding medications, providing a more comprehensive understanding of the relationship between impaired sleep and autoimmune disease risk.

## Materials and methods

### Study design and database

**FIGURE 1:**
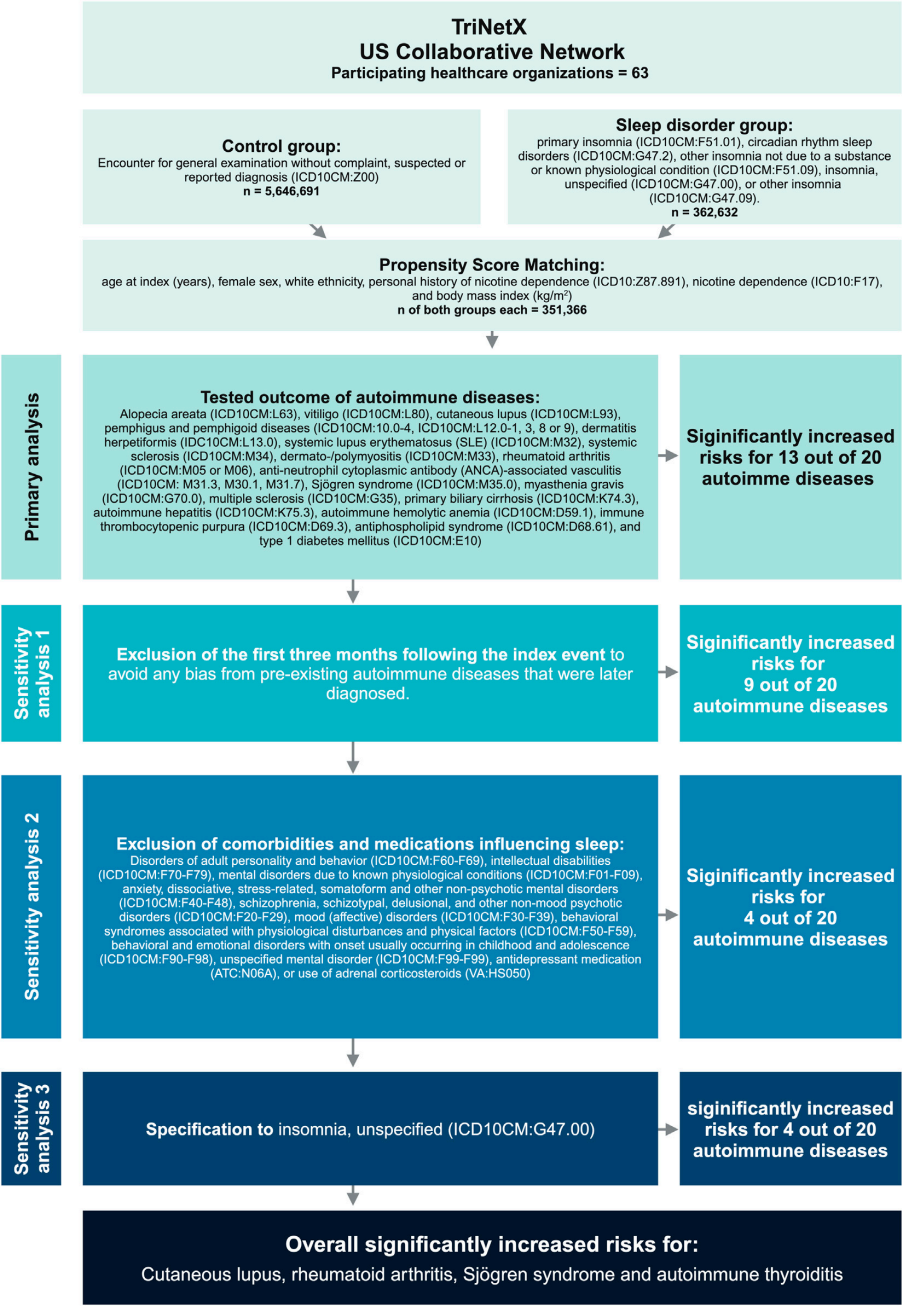
Study design. The database TriNetX was used to build cohorts of patients’ insomnia/circadian rhythm sleep disorders and healthy individuals. After propensity-matching (age, sex, ethnicity, and race) over 300,000 electronic health records were retrieved for each group. Next, the risk for subsequent diagnosis of any of the indicated autoimmune diseases, identified by their respective ICD10 code, was evaluated. To validate the approach, we also conducted several sensitivity analyses on the two populations, excluding confounders such as mental disorders or medications.

A global population-based retrospective cohort study with propensity-score matching was performed following previously published protocols (Kasperkiewicz et al., 2023; Olbrich et al., 2023; Ludwig et al., 2025). In detail, the US Collaborative Network of TriNetX was used to identify electronic health records (EHRs) with a diagnostic code for insomnia and non-insomnia controls. At the time of analysis, TriNetX provided access to over 120 million EHRs in five different networks. The US Collaborative Network was chosen as it includes the second largest number of EHRs and the most detailed information for each EHR among the different networks provided by TriNetX (Evans et al., 2021; Palchuk et al., 2023). Based on a collaboration between the University Clinic Schleswig-Holstein (UKSH) and TriNetX, UKSH researchers have access to the TriNetX database and analytical tools. In EHRs with a diagnostic code for insomnia and non-insomnia-controls the risk to develop any one of 20 selected autoimmune diseases was assessed. The study’s robustness was evaluated through three sensitivity analyses. In the first sensitivity analysis (S1), the initial 3 months following the index event were excluded to mitigate any bias arising from pre-existing autoimmune diseases that were later diagnosed. In the second sensitivity analysis (S2), exclusion criteria were applied throughout the entire follow-up period to ensure more stringent control. In the third sensitivity analysis (S3), the definition of insomnia was adjusted to address any potential bias arising from variations in the cohort definitions. Outcomes were defined prior to data acquisition and analysed after propensity-score matching. The study design is depicted in Figure 1. This figure shows the workflow from top to bottom. First, the groups were formed according to the respective International Classification of Disease, 10th Revision (ICD10) codes, thereafter, both groups were propensity score matched. Next, the primary analysis, testing for the 20 disorders followed. Below, the three sensitivity analyses are listed with the respective criteria. On the right, the outcomes of each step are listed and at the bottom of the figure, the overall result is described.

### Study population and definition of eligible patients

For the primary analysis and sensitivity analysis S1, the index event of the insomnia cohort was defined by the presence of primary insomnia (ICD10CM:F51.01; classified by disturbed sleep at least 3 nights per week for at least 3 months), circadian rhythm sleep disorders (ICD10CM:G47.2), other insomnia not due to a substance or known physiological condition (ICD10CM:F51.09), insomnia, unspecified (ICD10CM:G47.00), or other insomnia (ICD10CM:G47.09). EHRs indicating any of the following medications or diagnoses on or before the index event were excluded: Disorders of adult personality and behaviour (ICD10CM:F60-F69), intellectual disabilities (ICD10CM:F70-F79), MDs due to known physiological conditions (ICD10CM:F01-F09), anxiety, dissociative, stress-related, somatoform and other non-psychotic mental disorders (ICD10CM:F40-F48), schizophrenia, schizotypal, delusional, and other non-mood psychotic disorders (ICD10CM:F20-F29), mood (affective) disorders (ICD10CM:F30-F39), behavioural syndromes associated with physiological disturbances and physical factors (ICD10CM:F50-F59), behavioural and emotional disorders with onset usually occurring in childhood and adolescence (ICD10CM:F90-F98), unspecified mental disorder (ICD10CM:F99), antidepressant medication (ATC:N06A), or use of adrenal corticosteroids (VA:HS050). In the sensitivity analysis S2, above mentioned exclusion criteria had to be fulfilled before, on and 5 years after the index event. In sensitivity analysis S3, the index event of the insomnia cohort was defined by the presence of insomnia, unspecified (ICD10CM:G47.00). EHRs indicating any of the above-mentioned medications or diagnoses on or before the index event were excluded.

### Follow-up and definition of outcomes

In the primary analysis, as well as in sensitivity analyses S2 and S3, outcome within 5 years following the index event were considered. In the sensitivity analysis S1, outcomes occurring from 3 months to 5 years after the respective index events were considered. The following 20 autoimmune diseases were considered as outcomes: Alopecia areata (ICD10CM:L63), vitiligo (ICD10CM:L80), cutaneous lupus erythematosus (CLE) (ICD10CM:L93), pemphigus and pemphigoid diseases (ICD10CM:10.0-4, ICD10CM:L12.0-1, 3, 8 or 9), dermatitis herpetiformis (IDC10CM:L13.0), SLE (ICD10CM:M32), systemic sclerosis (SSc) (ICD10CM:M34), dermato-/polymyositis (ICD10CM:M33), RA (ICD10CM:M05 or M06), anti-neutrophil cytoplasmic antibody (ANCA)-associated vasculitis (AAV) (ICD10CM: M31.3, M30.1, M31.7), Sjögren syndrome (ICD10CM:M35.0), myasthenia gravis (ICD10CM:G70.0), multiple sclerosis (ICD10CM:G35), primary biliary cirrhosis (ICD10CM:K74.3), autoimmune hepatitis (ICD10CM:K75.3), autoimmune haemolytic anaemia (AHIA) (ICD10CM:D59.1), immune thrombocytopenic purpura (ICD10CM:D69.3), antiphospholipid syndrome (APS) (ICD10CM:D68.61), and type 1 diabetes mellitus (T1DM) (ICD10CM:E10). For all analyses, outcomes prior to the index event were excluded.

### Covariates

Propensity-score matching (PSM) is a method utilized in observational studies to balance cohorts by matching individuals based on their likelihood (propensity) of being assigned to a particular treatment or exposure group. This aims to weaken the influence of potential confounding variables, thus enhancing the comparability of the different groups. Several previous studies employed PSM to reduce potential biases inherent in observational research. PSM is potentially advantageous over conventional covariate adjustment methods (Elze et al., 2017). In this study, PSM was performed for each sub-analysis by establishing a covariate matrix. To account for potential risk factors confounding to the risk for autoimmune diseases, the following variables were included in the PSM: age at index (years, continuous), female sex (binary), white ethnicity (binary), personal history of nicotine dependence (ICD10:Z87.891, binary), nicotine dependence (ICD10:F17, binary), and body mass index (kg/m^2^, continuous). The matrix row order was randomized after data retrieval. A propensity-score for each patient was generated by logistic regression and matching was performed 1:1 using the greedy nearest neighbour approach with a calliper distance of 0.1 using the software package scikit-learn in Python (Hao and Ho, 2019; Pedregosa et al., 2011). Baseline characteristics were re-evaluated and reported after matching, differences were compared by t-test for continuous and z-test for binary or categorical variables.

### Statistical analysis

The statistical analysis was performed using the Survival package v3.2-3 in R (R Foundation for Statistical Computing, Vienna, Austria) and validated by comparison with the outputs of SAS version 9.4 (SAS, Cary, NC); graphs were created using GraphPad Prism (GraphPad Software Inc., Boston, MA). Relative risks and risk differences were calculated. Survival analyses were performed using the Kaplan-Meier method (KM). KM-curves were compared using the Log-rank test. A univariate Cox proportional hazards regression was used to express hazard ratios (HRs) with 95 %-confidence intervals (CIs). To counter the bias of multiple testing when investigating the risk to develop any of the 20 autoimmune diseases Bonferroni correction was applied (α = 0.05/20 = 0.0025).

## Results

### Cohort description

Prior to PSM, 362,632 cases with insomnia and 5,842,158 non-insomnia controls were identified. At the index event, insomnia cases had an average age of 52 ± 19.1 years, contrasting with controls at 41.1 ± 20.6 years (p < 0.0001, standardized difference 0.1490). Substantial differences were also evident across various demographic characteristics, comorbidities, and vital signs (Table 1). However, these disparities were either alleviated or lessened following PSM. Notably, the mean age after PSM was 52 ± 19.1 years for both groups, with no significant difference observed (p = 0.9980, standardized difference < 0.0001). Following PSM, a total of 351,366 EHRs were retained for both insomnia cases and non-insomnia controls (Table 1).

**TABLE 1:** Characteristics of patients with insomnia (cases) and non-insomnia controls before and after propensity-score matching (PSM) for the primary analysis.

Classification	Characteristic	Before matching		After matching	
		Insomnia	Controls	Insomnia	Controls
—	Number of participants	362,632	5,842,158	351,366	351,366
Demographics	Age at Index (years, SD)	52 ± 19.1	41.1 ± 20.6	52 ± 19.1	52 ± 19.1
	Female (%)	51.444	48.834	51.443	51.443
	*Male (%)*	45.372	45.625	45.373	44.162
	*Hispanic or Latino (%)*	7.4	10.485	7.401	8.298
	*Black or African American (%)*	12.34	13.713	12.34	11.896
	*Asian (%)*	3.973	4.419	3.973	3.517
	White (%)	63.304	54.324	63.303	63.304
Comorbidity	Nicotine dependence (%)	4.301	1.755	4.3	4.299
	Personal history of nicotine dependence (%)	3.022	1.257	3.02	3.021
Laboratory and vitals	BMI (kg/m^2^)	28.1 ± 6.5	27.4 ± 6.7	28.1 ± 6.5	28.2 ± 6.4

Items not included in PSM are shown in italic. Data was retrieved on March15th, 2024. *Abbreviations: BMI: Body mass index*.

### Insomnia is a risk factor for cutaneous lupus erythematosus, rheumatoid arthritis, Sjögren syndrome, and autoimmune thyroiditis

**FIGURE 2:**
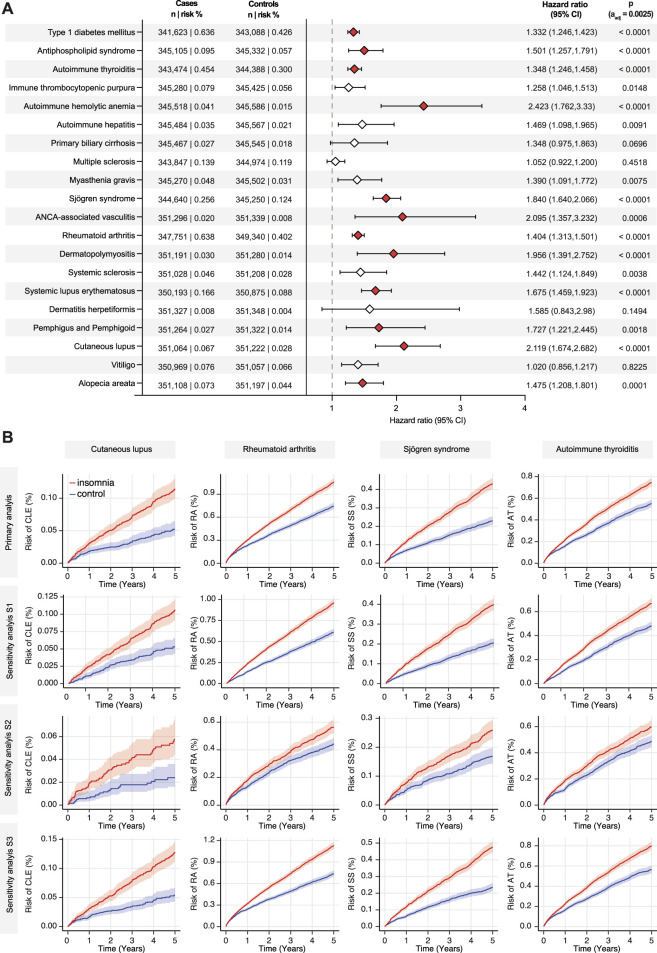
Insomnia is associated with an increased risk for cutaneous lupus, rheumatoid arthritis, Sjögren syndrome, and autoimmune thyroiditis. The TriNetX platform was queried to contrast the risk to develop any of 20 selected autoimmune diseases between individuals with a diagnostic code for insomnia and non-insomnia controls. In panel **(A)** all results of the primary analysis are depicted. Diamonds represent the hazard ratio (HR), whereas the error bars indicate the 95% confidence intervals (CI). Diamonds in red, indicate significantly increased HR in cases as opposed to non-insomnia controls. **(B)** Nelson-Allan plots of the indicated autoimmune diseases across the primary and all sensitivity analyses.

In the primary analysis and three sensitivity analyses (S1-S3), the risk of developing any of 20 selected autoimmune diseases was compared between insomnia cases and non-insomnia controls. While an elevated risk for the development of any of the chosen 20 diseases was observed in 13 of 20 autoimmune diseases in the primary analysis, this heightened risk persisted only for 4 out of 20 autoimmune diseases (CLE, RA, Sjögren syndrome, and autoimmune thyroiditis) across all sensitivity analyses. In detail, CLE was diagnosed in 0.067% of insomnia cases, compared to 0.028% in non-insomnia controls during the 5-year follow-up period. This difference in risk translated into a HR of 2.119 (95% CI 1.674-2.682, p < 0.0001). Notably, this heightened risk for CLE persisted in all three sensitivity analyses (S1-S3), with HR ranging from 1.956 to 2.369. Concerning RA, the risk of developing RA was 0.638% in EHRs indicating insomnia, compared to 0.402% in those without this diagnosis. This difference signified a slight but significantly increased risk for RA in individuals with insomnia (HR 1.404, CI 1.313-1.501, p < 0.0001). This risk, with similar HRs persisted in all sensitivity analyses (S1-S3). Following the index events, Sjögren syndrome was diagnosed in 0.256% of insomnia cases and in 0.124% of non-insomnia controls. Once more, this disparity corresponded to an elevated risk (HR 1.84, CI 1.64-2.066, p < 0.0001). This increased risk for Sjögren syndrome also persisted in all sensitivity analyses (S1-S3). Autoimmune thyroiditis was documented in 0.454% of insomnia cases and in 0.3% of non-insomnia controls. This discrepancy translated to an increased risk for autoimmune thyroiditis in individuals with insomnia, with a HR of 1.348 (CI 1.246-1.458, p < 0.0001). Moreover, this heightened risk persisted across all sensitivity analyses (S1-S3). These findings are depicted in Figure 2 and shown in detail in Supplementary Table 1.

### Insomnia is a potential risk factor for systemic lupus erythematosus, ANCA-associated vasculitis, autoimmune haemolytic anaemia, antiphospholipid syndrome and type 1 diabetes mellitus

In the primary analysis, an increased risk for SLE, AAV, AHIA, APS, and T1DM was also noted in those EHRs with a diagnostic code corresponding to insomnia as opposed to EHRs without indication thereof. Yet, these findings only persisted in two of the three sensitivity analyses. In detail, the risk for these diseases were found in sensitivity analyses S1 and S3, whilst in S2 these previous findings were not confirmed. In sensitivity analysis S2, psychiatric comorbidity was used as an exclusion criterion prior to the index event and throughout the entire follow-up period. In contrast, in all other analyses, psychiatric comorbidity only prior to the index event was used as exclusion criteria. Again, findings of the primary analysis are depicted in Figure 2 and shown in detail in Supplementary Table 1.

### Insomnia does not consistently alter the risk for 11 autoimmune diseases

As shown in Figure 2 and Supplementary Table 1, no or no persistent risk differences for alopecia areata, vitiligo, dermatitis herpetiformis, SSc, dermato-/polymyositis, myasthenia gravis, multiple sclerosis, primary biliary cirrhosis, autoimmune hepatitis, immune thrombocytopenic purpura, as well as for pemphigus and pemphigoid diseases were noted between EHRs indicating insomnia as opposed to those without.

## Discussion

In contrast to previous studies, which primarily examined adverse sleep patterns as a consequence of autoimmune diseases, this retrospective cohort study provides evidence that insomnia and CRSD significantly increase the risk of developing four distinct autoimmune diseases—CLE, RA, Sjögren syndrome, and autoimmune thyroiditis. These findings highlight the role of sleep disturbances as a potential contributing factor to autoimmunity through disruptions in multi-organ interactions, particularly within the autonomic nervous system, immune regulation, and neuroendocrine pathways. Sleep is a critical regulator of the HPA axis, and its disruption leads to dysregulated cortisol secretion, a key modulator of immune homeostasis (Balbo et al., 2010). Chronic hyperactivation of the HPA axis, as seen in insomnia, results in excessive glucocorticoid release, which can initially suppress inflammation but over time contributes to immune system dysregulation, increasing susceptibility to autoimmunity (Herman et al., 2016; Hirotsu et al., 2015; Bellavance and Rivest, 2014; Buckley et al., 2008).

This study employed extensive analyses to mitigate potential confounders, including psychiatric disorders, which can influence both sleep patterns and immune function. Interestingly, when psychiatric disorders and antidepressant use were not excluded, the risk of developing SLE, AAV, AHIA, APS, and T1DM also showed significant increases. This raises the question of whether psychiatric disorders, potentially through heightened systemic low-grade inflammation (Khandaker et al., 2017; Goldsmith et al., 2023; Osimo et al., 2019; Osimo et al., 2018), directly elevate the risk of autoimmunity or whether they exacerbate sleep disturbances, which then contribute to immune dysregulation. Chronic sleep impairment leads to persistent sympathetic nervous system activation, characterized by continuous catecholamine release (epinephrine and norepinephrine) (Greenlund and Carter, 2022; Dodt et al., 1997), which suppresses regulatory T-cell function while promoting pro-inflammatory cytokines such as Interleukin (IL-6), tumor necrosis factor (TNF), and IL-17 (Garbarino et al., 2021; Lange et al., 2022; Besedovsky et al., 2019)—key mediators in the pathogenesis of autoimmune diseases. Both mechanisms are plausible; however, this study directly implicates sleep disruption as an independent risk factor for autoimmune disease development.

Although this study reveals a significant link between disrupted sleep and increased autoimmunity risk, it is likely that the actual risk is underestimated. Insomnia and CRSD remain underreported, particularly among shift workers, such as those in the healthcare sector, where the importance of sleep quality is often overlooked and insufficiently addressed (Filip et al., 2017). Moreover, shift workers may perceive little benefit in seeking a diagnosis for a condition with a known occupational trigger, particularly when resolving the issue would require abandoning shift work—an economically unfeasible option for many. Similar challenges arise with sleep disturbances caused by social jetlag or caregiving responsibilities. Had these cases been included, the observed risks might have been even greater, potentially showing significance for additional autoimmune diseases. Furthermore, the prevalence of shift work is rising across various industries, including manufacturing, the food industry, and security sectors (Torquati et al., 2019; Wright et al., 2013). Given this growing trend, the impact of sleep disturbances on health demands heightened awareness among healthcare practitioners. Symptoms such as chronic exhaustion and fatigue should be carefully documented and taken seriously, as they may indicate an elevated risk for severe autoimmune conditions (Hsiao et al., 2015; Kuna et al., 2022; Stenger et al., 2023).

These findings emphasize the urgent need for further research into both the mechanisms underlying these associations and potential interventions. While eliminating shift work entirely is not feasible, optimizing shift schedules could mitigate its adverse effects. Studies suggest that forward-rotating shift schedules promote better circadian adaptation than backward-rotating schedules (d’Ettorre and Pellicani, 2020). Additionally, interventions such as melatonin supplementation may facilitate circadian realignment (Knauth and Hornberger, 2003), while exposure to bright light upon waking has been shown to counteract sleepiness and improve mood disorders (Pail et al., 2011; Al-Karawi and Jubair, 2016). Circadian misalignment, such as that seen in insomnia, affects not only melatonin secretion but also disrupts immune system rhythmicity. This desynchronization impacts leukocyte trafficking and nocturnal immune surveillance, leading to a pro-inflammatory state (Chertoff et al., 2022; Holtkamp et al., 2021; Inokawa et al., 2020) that primes the body for autoimmune reactivity. Conversely, minimizing blue light exposure before bedtime may further enhance sleep quality (Janků et al., 2020). Implementing these strategies could help reduce the detrimental effects of sleep disorders on overall health and autoimmune disease risk.

## Limitations

Several limitations of this study warrant consideration. First, the diagnoses and outcomes were defined solely based on ICD-10 codes without external validation, introducing the potential for misclassification. Second, the retrospective nature of the study and associated data collection methods pose inherent methodological challenges. To minimize bias, PSM and multiple sensitivity analyses were conducted. Third, the criteria used to define insomnia did not allow for assessing its severity, meaning that the impact of mild or moderate insomnia on autoimmune disease risk remains uncertain. It is possible that more severe cases were overrepresented in the dataset, further complicating risk estimation. Additionally, many individuals underreport sleep disturbances, particularly shift workers who may view their symptoms as unavoidable occupational hazards.

Moreover, psychiatric disorders are closely linked to sleep disturbances, yet these often go undiagnosed when attributed to underlying mental health conditions rather than primary sleep disorders. The fact that autoimmune diseases significantly associated with sleep disturbances lost statistical significance after excluding patients with psychiatric disorders suggests a potential interplay between mental health and autoimmunity. Sleep fragmentation and shortened sleep duration result in increased pro-inflammatory activity of the immune system, including sustained activation of the NF-κB pathway, which modulates stress responses and contributes to the persistence of autoimmune inflammation (Irwin et al., 2008; Barnabei et al., 2021). Another limitation concerns the prolonged diagnostic timeframe for certain autoimmune diseases, which can span several years or even a decade from initial symptom onset to an accurate diagnosis (Doria et al., 2010). This delay may further contribute to underestimating the impact of sleep disorders on autoimmunity development. Additionally, this study did not account for genetic predisposition or family history of autoimmune diseases, both of which could influence individual susceptibility.

A further consideration is the inclusion of patients using sedative medications. While excluding these individuals would have significantly reduced the sample size and limited statistical power, the role of sedatives in immune modulation must be acknowledged. Some medications, such as melatonin receptor agonists and orexin receptor antagonists, appear to have minimal effects on immune function (Meng et al., 2022). Others, such as alpha-2 adrenoceptor agonists, may exert pro-inflammatory effects that could theoretically enhance autoimmunity (Sanders et al., 2009). Conversely, benzodiazepines and other sedatives with anti-inflammatory properties could attenuate systemic inflammation and potentially reduce autoimmune disease risk (Sanders et al., 2009; Villa et al., 2018). Thus, including patients on sleep medications likely did not lead to an overestimation of the observed effects; if anything, it may have resulted in a conservative estimate of the true risk.

## Conclusion

Despite these limitations, this large-scale population-based study demonstrates a significant association between insomnia and the development of four autoimmune diseases. When patients with psychiatric disorders were not excluded, the number of autoimmune diseases significantly linked to sleep disturbances increased to nine. Given that lifestyle and socioeconomic factors such as shift work contribute to chronic sleep impairment, these findings underscore the importance of recognizing sleep disorders as a serious health concern. Chronic sleep disturbances are not merely symptoms of autoimmune diseases but may act as catalysts by altering neuroendocrine and immune system balance, promoting systemic inflammation and immune dysfunction. Clinicians should be vigilant in assessing and addressing sleep disturbances, particularly in patients at risk for autoimmune diseases. Moving forward, additional research is necessary to fully elucidate the interactions between sleep disorders, mental health conditions, and immune dysregulation. These data highlight insomnia as a substantial risk factor for specific autoimmune diseases, suggesting that effective treatment and prevention of sleep disturbances may have far-reaching implications for reducing autoimmune disease burden.

## Data Availability

The original contributions presented in the study are included in the article/Supplementary Material, further inquiries can be directed to the corresponding author.
