# Supplementary material for: Insomnia increases the risk for specific autoimmune diseases: a large-scale retrospective cohort study
Source: Front Netw Physiol. 2025 Apr 10;5:1499297. doi: 10.3389/fnetp.2025.1499297 (PMC12018472; doi:10.3389/fnetp.2025.1499297)
Supplement: Supplementary file 1 [file Table1.docx]

| **Outcome** | **Analysis** | **Insomnia**  **(Cases)** | | | **Non-insomnia controls** | | | **Results** | | | | |
| --- | --- | --- | --- | --- | --- | --- | --- | --- | --- | --- | --- | --- |
|  |  | **N of eligible participants** | **N of Out-comes** | **Risk, %** | **N of eligible participants** | **N of Out-comes** | **Risk, %** | **Risk difference, %** | **(95% confidence interval)** | **Hazard ratio** | **(95% confidence interval)** | **P value**  **P value**  **α_adj_=0.0025** |
| **Alopecia areata** | **Primary** | 351,108 | 258 | 0.073 | 351,197 | 154 | 0.044 | 0.03 | (0.018%,0.041%) | **1.475** | (1.208,1.801) | **0.0001** |
|  | **S1** | 345,345 | 222 | 0.064 | 345,434 | 142 | 0.041 | 0.023 | (0.012%,0.034%) | 1.373 | (1.112,1.695) | 0.0031 |
|  | **S2** | 210,110 | 76 | 0.036 | 210,193 | 72 | 0.034 | 0.002 | (-0.009%,0.013%) | 1.011 | (0.732,1.395) | 0.9475 |
|  | **S3** | 316,617 | 249 | 0.079 | 316,709 | 154 | 0.049 | 0.03 | (0.018%,0.042%) | **1.403** | (1.148,1.716) | **0.0009** |
| **Vitiligo** | **Primary** | 350,969 | 268 | 0.076 | 351,057 | 231 | 0.066 | 0.011 | (-0.002%,0.023%) | 1.02 | (0.856,1.217) | 0.8225 |
|  | **S1** | 345,208 | 235 | 0.068 | 345,275 | 173 | 0.05 | 0.018 | (0.007%,0.029%) | 1.194 | (0.981,1.453) | 0.0762 |
|  | **S2** | 210,040 | 103 | 0.049 | 210,109 | 125 | 0.059 | -0.01 | (-0.025%,0.004%) | 0.79 | (0.609,1.025) | 0.0759 |
|  | **S3** | 316,483 | 265 | 0.084 | 316,576 | 178 | 0.056 | 0.028 | (0.014%,0.041%) | 1.291 | (1.067,1.561) | 0.0083 |
| **Cutaneous lupus erythematosus** | **Primary** | 351,064 | 236 | 0.067 | 351,222 | 98 | 0.028 | 0.039 | (0.029%,0.05%) | **2.119** | (1.674,2.682) | **< 0.0001** |
|  | **S1** | 345,304 | 207 | 0.06 | 345,480 | 93 | 0.027 | 0.033 | (0.023%,0.043%) | **1.956** | (1.531,2.498) | **< 0.0001** |
|  | **S2** | 210,122 | 64 | 0.03 | 210,200 | 26 | 0.012 | 0.018 | (0.009%,0.027%) | **2.369** | (1.502,3.738) | **0.0001** |
|  | **S3** | 316,564 | 240 | 0.076 | 316,712 | 93 | 0.029 | 0.046 | (0.035%,0.058%) | **2.233** | (1.757,2.837) | **< 0.0001** |
| **Pemphigus and Pemphigoid** | **Primary** | 351,264 | 95 | 0.027 | 351,322 | 48 | 0.014 | 0.013 | (0.007%,0.02%) | **1.727** | (1.221,2.445) | **0.0018** |
|  | **S1** | 345,521 | 84 | 0.024 | 345,566 | 51 | 0.015 | 0.01 | (0.003%,0.016%) | 1.435 | (1.013,2.032) | 0.0409 |
|  | **S2** | 210,221 | 29 | 0.014 | 210,253 | 25 | 0.012 | 0.002 | (-0.005%,0.009%) | 1.106 | (0.648,1.888) | 0.7121 |
|  | **S3** | 316,756 | 95 | 0.03 | 316,814 | 63 | 0.02 | 0.01 | (0.002%,0.018%) | 1.293 | (0.941,1.779) | 0.1125 |
| **Dermatitis herpetiformis** | **Primary** | 351,327 | 27 | 0.008 | 351,348 | 15 | 0.004 | 0.003 | (0%,0.007%) | 1.585 | (0.843,2.98) | 0.1494 |
|  | **S1** | 345,589 | 21 | 0.006 | 345,608 | 19 | 0.005 | 0.001 | (-0.003%,0.004%) | 0.977 | (0.525,1.818) | 0.9422 |
|  | **S2** | 210,255 | 10 | 0.005 | 210,267 | 10 | 0.005 | NaN | (-0.004%,0.004%) | 0.598 | (0.196,1.828) | 0.3618 |
|  | **S3** | 316,813 | 26 | 0.008 | 316,835 | 18 | 0.006 | 0.003 | (-0.002%,0.007%) | 1.267 | (0.695,2.312) | 0.4389 |
| **Systemic lupus erythematosus** | **Primary** | 350,193 | 583 | 0.166 | 350,875 | 309 | 0.088 | 0.078 | (0.062%,0.095%) | **1.675** | (1.459,1.923) | **< 0.0001** |
|  | **S1** | 344,368 | 469 | 0.136 | 345,113 | 257 | 0.074 | 0.062 | (0.046%,0.077%) | **1.61** | (1.383,1.874) | **< 0.0001** |
|  | **S2** | 209,710 | 151 | 0.072 | 210,009 | 101 | 0.048 | 0.024 | (0.009%,0.039%) | 1.433 | (1.114,1.844) | 0.0049 |
|  | **S3** | 315,736 | 564 | 0.179 | 316,404 | 288 | 0.091 | 0.088 | (0.07%,0.106%) | **1.717** | (1.489,1.979) | **< 0.0001** |
| **Systemic sclerosis** | **Primary** | 351,028 | 163 | 0.046 | 351,208 | 100 | 0.028 | 0.018 | (0.009%,0.027%) | 1.442 | (1.124,1.849) | 0.0038 |
|  | **S1** | 345,273 | 135 | 0.039 | 345,464 | 84 | 0.024 | 0.015 | (0.006%,0.023%) | 1.417 | (1.079,1.861) | 0.0118 |
|  | **S2** | 210,120 | 50 | 0.024 | 210,186 | 38 | 0.018 | 0.006 | (-0.003%,0.014%) | 1.258 | (0.825,1.918) | 0.2854 |
|  | **S3** | 316,532 | 166 | 0.052 | 316,691 | 95 | 0.03 | 0.022 | (0.012%,0.032%) | **1.519** | (1.181,1.955) | **0.0011** |
| **Dermato-/polymyositis** | **Primary** | 351,191 | 106 | 0.03 | 351,280 | 48 | 0.014 | 0.017 | (0.01%,0.023%) | **1.956** | (1.391,2.752) | **< 0.0001** |
|  | **S1** | 345,443 | 88 | 0.025 | 345,552 | 65 | 0.019 | 0.007 | (0%,0.014%) | 1.189 | (0.863,1.638) | 0.2899 |
|  | **S2** | 210,180 | 24 | 0.011 | 210,240 | 30 | 0.014 | -0.003 | (-0.01%,0.004%) | 0.769 | (0.45,1.316) | 0.3364 |
|  | **S3** | 316,686 | 102 | 0.032 | 316,764 | 63 | 0.02 | 0.012 | (0.004%,0.02%) | 1.419 | (1.036,1.943) | 0.0282 |
| **Rheumatoid arthritis** | **Primary** | 347,751 | 2,220 | 0.638 | 349,340 | 1,403 | 0.402 | 0.237 | (0.203%,0.271%) | **1.404** | (1.313,1.501) | **< 0.0001** |
|  | **S1** | 341,723 | 1,880 | 0.55 | 343,333 | 1,042 | 0.303 | 0.247 | (0.216%,0.278%) | **1.595** | (1.478,1.72) | **< 0.0001** |
|  | **S2** | 208,451 | 624 | 0.299 | 209,250 | 475 | 0.227 | 0.072 | (0.041%,0.103%) | **1.266** | (1.123,1.426) | **0.0001** |
|  | **S3** | 313,395 | 2,181 | 0.696 | 314,906 | 1,281 | 0.407 | 0.289 | (0.253%,0.326%) | **1.493** | (1.393,1.599) | **< 0.0001** |
| **ANCA-associated vasculitis** | **Primary** | 351,296 | 69 | 0.02 | 351,339 | 29 | 0.008 | 0.011 | (0.006%,0.017%) | **2.095** | (1.357,3.232) | **0.0006** |
|  | **S1** | 345,550 | 55 | 0.016 | 345,594 | 14 | 0.004 | 0.012 | (0.007%,0.017%) | **3.454** | (1.921,6.211) | **< 0.0001** |
|  | **S2** | 210,249 | 12 | 0.006 | 210,258 | 10 | 0.005 | 0.001 | (-0.003%,0.005%) | 1.288 | (0.543,3.056) | 0.5654 |
|  | **S3** | 316,786 | 68 | 0.021 | 316,808 | 30 | 0.009 | 0.012 | (0.006%,0.018%) | **1.98** | (1.289,3.044) | **0.0015** |
| **Sjögren syndrome** | **Primary** | 344,640 | 884 | 0.256 | 345,250 | 427 | 0.124 | 0.133 | (0.112%,0.153%) | **1.84** | (1.64,2.066) | **< 0.0001** |
|  | **S1** | 344,543 | 787 | 0.228 | 345,181 | 358 | 0.104 | 0.125 | (0.105%,0.144%) | **1.94** | (1.712,2.198) | **< 0.0001** |
|  | **S2** | 204,216 | 271 | 0.133 | 204,501 | 172 | 0.084 | 0.049 | (0.028%,0.069%) | **1.519** | (1.255,1.839) | **< 0.0001** |
|  | **S3** | 315,883 | 898 | 0.284 | 316,453 | 400 | 0.126 | 0.158 | (0.136%,0.18%) | **1.949** | (1.732,2.193) | **< 0.0001** |
| **Myasthenia gravis** | **Primary** | 345,270 | 167 | 0.048 | 345,502 | 107 | 0.031 | 0.017 | (0.008%,0.027%) | 1.39 | (1.091,1.772) | 0.0075 |
|  | **S1** | 345,245 | 142 | 0.041 | 345,476 | 81 | 0.023 | 0.018 | (0.009%,0.026%) | 1.545 | (1.176,2.03) | **0.0016** |
|  | **S2** | 204,511 | 51 | 0.025 | 204,651 | 28 | 0.014 | 0.011 | (0.003%,0.02%) | 1.752 | (1.105,2.778) | 0.0157 |
|  | **S3** | 316,503 | 159 | 0.05 | 316,733 | 95 | 0.03 | 0.02 | (0.01%,0.03%) | 1.465 | (1.136,1.889) | 0.0031 |
| **Multiple sclerosis** | **Primary** | 343,847 | 479 | 0.139 | 344,974 | 411 | 0.119 | 0.02 | (0.003%,0.037%) | 1.052 | (0.922,1.2) | 0.4518 |
|  | **S1** | 343,753 | 385 | 0.112 | 344,843 | 280 | 0.081 | 0.031 | (0.016%,0.045%) | 1.225 | (1.05,1.429) | 0.0098 |
|  | **S2** | 203,819 | 135 | 0.066 | 204,356 | 119 | 0.058 | 0.008 | (-0.007%,0.023%) | 1.096 | (0.857,1.403) | 0.4639 |
|  | **S3** | 315,245 | 443 | 0.141 | 316,229 | 406 | 0.128 | 0.012 | (-0.006%,0.03%) | 0.965 | (0.844,1.105) | 0.6093 |
| **Primary biliary cirrhosis** | **Primary** | 345,467 | 92 | 0.027 | 345,545 | 61 | 0.018 | 0.009 | (0.002%,0.016%) | 1.348 | (0.975,1.863) | 0.0696 |
|  | **S1** | 345,443 | 68 | 0.02 | 345,532 | 48 | 0.014 | 0.006 | (0%,0.012%) | 1.249 | (0.863,1.807) | 0.2381 |
|  | **S2** | 204,630 | 27 | 0.013 | 204,656 | 22 | 0.011 | 0.002 | (-0.004%,0.009%) | 1.183 | (0.674,2.077) | 0.5582 |
|  | **S3** | 316,685 | 87 | 0.027 | 316,757 | 54 | 0.017 | 0.01 | (0.003%,0.018%) | 1.414 | (1.007,1.986) | 0.0445 |
| **Autoimmune hepatitis** | **Primary** | 345,484 | 120 | 0.035 | 345,567 | 73 | 0.021 | 0.014 | (0.006%,0.021%) | 1.469 | (1.098,1.965) | 0.0091 |
|  | **S1** | 345,449 | 85 | 0.025 | 345,549 | 55 | 0.016 | 0.009 | (0.002%,0.015%) | 1.357 | (0.967,1.906) | 0.0765 |
|  | **S2** | 204,649 | 27 | 0.013 | 204,664 | 21 | 0.01 | 0.003 | (-0.004%,0.01%) | 1.237 | (0.699,2.187) | 0.4646 |
|  | **S3** | 316,706 | 115 | 0.036 | 316,772 | 62 | 0.02 | 0.017 | (0.009%,0.025%) | 1.635 | (1.2,2.226) | **0.0016** |
| **Autoimmune haemolytic anaemia** | **Primary** | 345,518 | 141 | 0.041 | 345,586 | 52 | 0.015 | 0.026 | (0.018%,0.034%) | **2.423** | (1.762,3.33) | **< 0.0001** |
|  | **S1** | 345,485 | 108 | 0.031 | 345,576 | 42 | 0.012 | 0.019 | (0.012%,0.026%) | **2.262** | (1.584,3.231) | **< 0.0001** |
|  | **S2** | 204,667 | 25 | 0.012 | 204,691 | 13 | 0.006 | 0.006 | (0%,0.012%) | 1.838 | (0.94,3.592) | 0.0708 |
|  | **S3** | 316,741 | 135 | 0.043 | 316,809 | 58 | 0.018 | 0.024 | (0.016%,0.033%) | **2.045** | (1.503,2.783) | **< 0.0001** |
| **Immune thrombocytopenic purpura** | **Primary** | 345,280 | 272 | 0.079 | 345,425 | 192 | 0.056 | 0.023 | (0.011%,0.035%) | 1.258 | (1.046,1.513) | 0.0148 |
|  | **S1** | 345,232 | 224 | 0.065 | 345,391 | 158 | 0.046 | 0.019 | (0.008%,0.03%) | 1.244 | (1.015,1.526) | 0.0350 |
|  | **S2** | 204,517 | 72 | 0.035 | 204,620 | 67 | 0.033 | 0.002 | (-0.009%,0.014%) | 1.034 | (0.742,1.443) | 0.8423 |
|  | **S3** | 316,518 | 280 | 0.088 | 316,688 | 155 | 0.049 | 0.04 | (0.027%,0.052%) | **1.569** | (1.29,1.91) | **< 0.0001** |
| **Autoimmune thyroiditis** | **Primary** | 343,474 | 1,559 | 0.454 | 344,388 | 1,032 | 0.3 | 0.154 | (0.125%,0.183%) | **1.348** | (1.246,1.458) | **< 0.0001** |
|  | **S1** | 343,241 | 1,326 | 0.386 | 344,189 | 833 | 0.242 | 0.144 | (0.118%,0.171%) | **1.407** | (1.29,1.534) | **< 0.0001** |
|  | **S2** | 203,403 | 636 | 0.313 | 203,960 | 491 | 0.241 | 0.072 | (0.04%,0.104%) | **1.251** | (1.112,1.408) | **0.0002** |
|  | **S3** | 314,845 | 1,553 | 0.493 | 315,606 | 974 | 0.309 | 0.185 | (0.153%,0.216%) | **1.394** | (1.287,1.51) | **< 0.0001** |
| **Antiphospholipid syndrome** | **Primary** | 345,105 | 328 | 0.095 | 345,332 | 196 | 0.057 | 0.038 | (0.025%,0.051%) | **1.501** | (1.257,1.791) | **< 0.0001** |
|  | **S1** | 345,049 | 272 | 0.079 | 345,291 | 155 | 0.045 | 0.034 | (0.022%,0.046%) | **1.558** | (1.279,1.897) | **< 0.0001** |
|  | **S2** | 204,456 | 92 | 0.045 | 204,547 | 70 | 0.034 | 0.011 | (-0.001%,0.023%) | 1.272 | (0.932,1.735) | 0.1289 |
|  | **S3** | 316,348 | 318 | 0.101 | 316,554 | 210 | 0.066 | 0.034 | (0.02%,0.048%) | 1.33 | (1.117,1.583) | **0.0013** |
| **Type 1 diabetes mellitus** | **Primary** | 341,623 | 2,173 | 0.636 | 343,088 | 1,462 | 0.426 | 0.21 | (0.176%,0.244%) | **1.332** | (1.246,1.423) | **< 0.0001** |
|  | **S1** | 341,266 | 1,816 | 0.532 | 342,812 | 1,186 | 0.346 | 0.186 | (0.155%,0.218%) | **1.359** | (1.263,1.462) | **< 0.0001** |
|  | **S2** | 202,712 | 640 | 0.316 | 203,311 | 613 | 0.302 | 0.014 | (-0.02%,0.048%) | 1.009 | (0.903,1.127) | 0.8745 |
|  | **S3** | 313,135 | 2,051 | 0.655 | 314,463 | 1,324 | 0.421 | 0.234 | (0.198%,0.27%) | **1.363** | (1.272,1.46) | **< 0.0001** |

**Supplement Tab. 1**. Risk of autoimmune diseases in patients with insomnia. Electronic health records (EHRs) from the US Collaborative Network of the TriNetX database including data from 63 healthcare institutes about 20 autoimmune diseases influenced by sleep disorders. Significant findings are highlighted in bold. Data was analysed on March15th, 2024.
